# Integrating Maternal and Child Health Into Climate Change: A Holistic Approach

**DOI:** 10.3389/phrs.2024.1607553

**Published:** 2025-01-03

**Authors:** Felix Amekpor, Waheed Sakariyau, Nathan Ezie Kengo, Nwodo Amarachukwu Sandra, Joseph Agyapong, Zakariya’u Dauda, Samuel Kwarteng, David Adeoye Adedokun, Gideon Darko

**Affiliations:** ^1^ Noguchi Memorial Institute for Medical Research, University of Ghana, Accra, Ghana; ^2^ College of Health and Natural Science, The University of Tulsa, Tulsa, OK, United States; ^3^ Faculty of Medicine and Biomedical Sciences, University of Garoua, Garoua, Cameroon; ^4^ Department of Microbiology, University of Nigeria, Nsukka, Nsukka, Nigeria; ^5^ Department of Microbiology and Immunology, Kwame Nkrumah University of Science and Technology, Kumasi, Ghana; ^6^ School of Medical Laboratory Science, Usmanu Danfodiyo University, Sokoto, Nigeria; ^7^ Department of Molecular Medicine, Kwame Nkrumah University of Science and Technology, Kumasi, Ghana; ^8^ West African Centre for Cell Biology of Infectious Pathogens, College of Basic and Applied Sciences, University of Ghana, Accra, Ghana; ^9^ Faculty of Agriculture, Kwame Nkrumah University of Science and Technology, Kumasi, Ghana

**Keywords:** heat, malnutrition, forced migration, infectious diseases, mental health problems

## Abstract

**Objectives:**

In everyday language, climate change is an increase in the Earth’s average temperature. Climate change negatively affects life support systems, including air, food, water, shelter, and security, on which humans depend. This paper aims to holistically integrate maternal and child health into climate change.

**Methods:**

A narrative/literature review approach were adopted using papers sources from google scholar, research gate and web of science. About 10 papers was initially gathered and it was later scrutinized to 6.

**Results:**

It was discovered that, climate change negatively impacts food and water security, heat stress, extreme weather, and air pollution, with women and children most affected. The World Health Organization estimates 250,000 climate-related deaths annually by 2050, disproportionately affecting maternal and child health. Integrating climate and maternal health strategies could offer benefits, yet research on adapting to climate change’s effects on pregnancy outcomes is limited.

**Conclusion:**

Addressing maternal and child health requires integrating health-focused strategies into environmental policies to reduce vulnerabilities to climate-related risks. A comprehensive approach can enhance resilience by improving healthcare access, education, and sustainable resource management, benefiting public health and environmental outcomes.

## Introduction

Climate change, recognized as one of the most significant global threats of the 21st century, is associated with immediate harm to early childhood development, long-term health consequences, and profound effects on future generations. These impacts extend beyond the present, influencing not only current health outcomes but also shaping the wellbeing of future populations [1]. To protect vulnerable populations, the relationship between climate change and mother and child health is essential. Human activity-induced climate change has accelerated the development of vector-borne illnesses, raising health hazards for young children and expectant mothers [1]. Furthermore, the paucity of resources brought on by climate change increases susceptibility to malnutrition and associated health issues. The World Health Organization and other international health organizations stress how critical it is to understand the unique health risks that pregnant women and children confront considering the shifting environment [2]. A holistic strategy must incorporate interventions, fortify health systems, foster community resilience, and advance sustainable development objectives. Therefore, understanding the complex pathways through which environmental shifts impact vulnerable populations is necessary for a comprehensive approach to addressing maternal and child health in the context of climate change mitigation. The global community’s commitment to achieving the Sustainable Development Goals (SDGs) underscores the need for a synergistic approach addressing maternal and child health while mitigating climate change.

## Methods

In order to thoroughly examine the body of literature already written about the research issue, this review paper employed a narrative review methodology. The procedure started with a thorough search of pertinent scholarly articles from three important databases: Web of Science, ResearchGate, and Google Scholar. These platforms were picked in order to guarantee that scholarly sources of a high caliber and variety would be included.

Ten papers were first selected based on their titles, abstracts, and applicability to the research question. Studies that were published in peer-reviewed publications and that addressed important issues about how climate change affects the health of mothers and children were among the selection criteria for inclusion. Furthermore, only English-language works published in the previous 10 years were used, guaranteeing the literature’s currentness and relevancy.

A thorough screening procedure was applied to every document after it was first collected. The quality, methodology, and conclusions of the papers were evaluated by full-text reviews. Studies that examined the connection between climate change and its consequences on pregnancy outcomes—more especially, mother and child health—were the subject of further refinement of the inclusion criteria.

Six papers were ultimately included after a rigorous examination. These studies were selected based on their methodological soundness, relevance to the larger framework of how climate change affects mother and child health outcomes, and compatibility with the research topic. These criteria were not met, and papers that were judged to be overly vague or out-of-date were not included in the final selection.

In order to minimize any bias and provide a clear and thorough grasp of the subject, this systematic methodology made sure that the review concentrated on the most pertinent and trustworthy studies.

## Results

### Pregnancy Risks in Changing Climates

**FIGURE 1:**
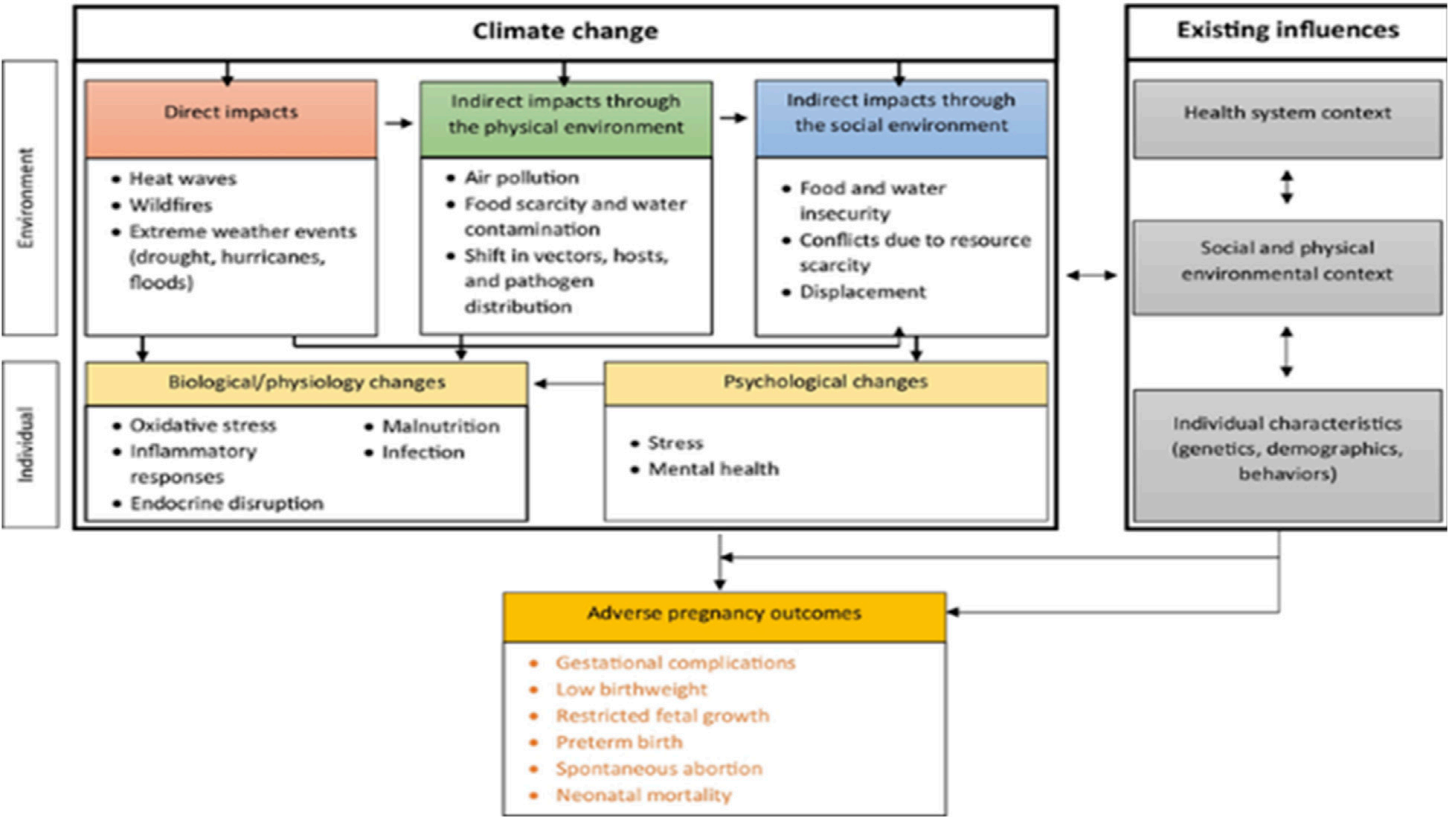
Implications of climate change on pregnancy outcomes [5] (Accra, Ghana. 2024).

An estimated 2.0 million stillbirths, 2.5 million neonatal deaths, and 295,000 maternal fatalities occur annually worldwide [1]. Pregnancy and labor are major health risks in low-income nations, with maternal morbidity and maternal deaths being more prevalent in these countries [2]. Climate change is one of the largest threats to world health in the 21st century [3], with long-term effects on pregnant mothers, infants, and future generations. Pregnant women and newborns are increasingly recognized as vulnerable groups, and extreme weather events like heat waves, droughts, and storms are affecting their health [2, 4]. Climate change can impact pregnancy health directly through specific climatic disasters and indirectly through changes to natural and social environments. Pregnancy-related disasters are linked to higher risks of prenatal problems, pregnancy loss, reduced fetal growth, low birth weight, preterm birth, and specific delivery/newborn issues [4]. Despite the controlled physiological and psychological changes experienced by pregnant mothers and the developing fetus, there is limited research on how to adapt to and reduce the effects of climate change on pregnancy outcomes. The consequences of the climate on the health of expectant mothers can be recognized as (a) direct impacts via discrete environmental disasters [5], (b) indirect impacts through changes in the natural environment, and (c) indirect impacts through changes in the social environment (Figure 1).

### Implications of Changing Climate on Pregnancy Outcomes

Physiological and anatomical changes that occur during pregnancy and the newborn period reduce the body’s capacity to regulate body temperature. Pregnant women experience significant body changes, including increased body mass index, fat deposition, and increased metabolism due to fetal growth [5, 6]. Heat stress, resulting from the inability to maintain thermal balance, releases heat-shock proteins, which can negatively impact the health of expectant mothers and their babies. Heat exposure is linked to risks of stillbirth, low birth weight, premature membrane rupture, and preterm birth. Infants with low birth weight are more vulnerable to infections [7]. Dehydration caused by increased sweating during pregnancy can cause labor to start prematurely and persist for longer. Prenatal exposure to extreme weather events like heat waves, wildfires, and droughts has been linked to adverse pregnancy outcomes [6].

### Childhood Diseases and Climate

Global climate change (CC) is widely regarded as the most significant threat to human health in the 21st century, impacting air quality, food security, water resources, and disease prevalence. Due to their developing organ systems, psychological immaturity, the nature of their everyday activities, and higher levels of exposure, children are especially the major victims of climate catastrophe [8–10]. Data suggests that climate change is contributing to the rise in certain disorders and allergies by intensifying environmental factors, such as air pollution, allergen exposure, and extreme weather events, which interact with genetic predispositions to exacerbate health outcomes. A child’s development can be greatly impacted by the complex interactions between climate change and childhood diseases, which can lead to stress, mental health issues, food-borne, water-borne, or vector-borne diseases, respiratory disorders, malnutrition, illnesses brought on by extreme heat, and displacement [9]. According to the British Thoracic Society’s report, *The Environment and Lung Health 2020*, individuals with chronic respiratory conditions are particularly vulnerable to the adverse effects of climate change, as environmental changes exacerbate respiratory health challenges. Due to their smaller peripheral airways, developing bodies, and higher breathing rates, children, especially those under the age of five—are more vulnerable than adults to CC-related respiratory morbidity [10].

Additionally, during the warmer months, youngsters typically play outside more. Consequently, because of their limited capacity to sustain ideal internal temperatures during heat stress, they are more vulnerable to the harmful effects of exposure to high temperatures [10]. A child born today will grow up in a world projected to be over four degrees warmer than pre-industrial levels, with climate change impacting their health throughout their lifetime, from birth through old age [11]. Unpredictable precipitation patterns driven by climate change have been linked to an increase in communicable and vector-borne diseases, as altered rainfall influences waterborne pathogens’ spread and expands habitats for vectors like mosquitoes [12]. Heat waves have been connected to reduced cognitive function and an increase in mortality while maternal stress, nutritional insults from low harvests, and exposure to infectious diseases have all been associated with poor childhood growth and development because of climate change [7, 13]. Pediatricians need to incorporate evidence-based knowledge into their clinical practice and fully understand the effects of climate change (CC) on children’s health to offer comprehensive care. This is crucial, especially considering their responsibility to educate families on the implications of CC and its impact on children’s wellbeing [10].

### Maternal Health

The World Health Organization (WHO) defines maternal health as the wellbeing of women during pregnancy, childbirth, and the postpartum period [14]. Approximately 536,000 women die annually due to complications, with 99% occurring in developing countries. The UN Millennium Development Goals (MDGs) aim to address maternal health, with MDG 5 aiming for a 75% reduction in maternal mortality and universal access to reproductive health [15]. MDG 4 aims to reduce child mortality by two-thirds by 2015, addressing prevalent causes like pneumonia, diarrhea, and malaria in children under five. Climate change poses a significant threat to achieving MDG 5, necessitating a more nuanced understanding of its impact on maternal health [15, 16]. Migrant birth outcomes are also affected by forced resettlement, population movements, and inadequate medical services. The COVID-19 pandemic has highlighted the potential for disruptions in low- and middle-income countries to result in additional maternal and child deaths [17, 18].

### Pediatric Health Interventions

Over the past decades, efforts have been made to achieve the Millennium Development Goal (MDG) on child survival, with 13 countries experiencing an increase in overall child deaths [19]. However, some have met the MDG target of reducing mortality rates among children under five by two-thirds. In 2020, around 5 million children under five died, mostly from treatable and preventable causes [20]. Newborns in the first 28 days of life accounted for approximately 2.4 million of these deaths. Child mortality in sub-Saharan Africa (SSA) remained the highest in the world at 74 deaths per 1,000 live births, 14 times higher than the risk for children in Europe and North America [20].

Preterm birth problems, birth asphyxia/trauma, pneumonia, diarrhea, and malaria are the major causes of death for children under the age of five. Access to health and sanitation treatments can help avoid or treat these conditions [21]. However, most child health programs do not reach the world’s poorest families, contributing to the inability to meet proposed reductions in child mortality numbers [20, 22]. The SDG agenda emphasizes the need for inclusive and targeted strategies for the most vulnerable and marginalized children. Evidence suggests that effective interventions in pregnancy and delivery care, hygiene facilities, environmental settings, and access to health resources can improve child survival and health [23]. Vaccination campaigns and other survival interventions have shown that the children who need it most are not adequately covered. Initiatives such as the measles vaccination campaign have reduced measles mortality in sub-Saharan Africa by 92% between 2000 and 2008. Implementing WHO guidelines on the management of severe acute malnutrition has also shown a reduction in child mortality rates [20, 24].

### Long-Term Developmental Effects

In everyday language, climate change refers to the ongoing increase in the Earth’s average temperature, commonly known as global warming, and its repercussions on the planet’s climate system [1]. This phenomenon is giving rise to a spectrum of escalating environmental impacts. Even if endeavors to curtail future warming prove successful, certain consequences will persist for centuries, including ocean heating, ocean acidification, and rising sea levels [2]. The impact of global warming on local climates varies across regions, countries, and specific locations [4]. The Arctic region is expected to experience a significant temperature rise, with the potential human health impact expected to be minor due to initial low temperatures [5]. However, the “urban heat island effect” and decline in air quality in tropical regions can create hot spots, posing additional risks. Climate change can affect human health through issues like insufficient access to food, safe drinking water, substandard sanitation, population displacement, disease patterns, extreme weather events, and lack of shelter [25, 26]. Maternal health is crucial, with approximately 536,000 women dying annually due to pregnancy, childbirth, or postpartum complications. Blood loss or hypertension accounts for half of all maternal deaths, while indirect causes like malaria, HIV/AIDS, and heart diseases contribute to 18% and 11%, respectively [5, 11]. Most maternal deaths are preventable, with 99% occurring in developing countries. These factors collectively heighten susceptibility to variations in external temperatures, sometimes leading to significant alterations in core body temperature [6]. Table 1 delineates specific pregnancy complications with a focus on climate-related factors.

**TABLE 1:** Instances of clinical and physiological consequences of pregnancy resulting from hormonal shifts (Accra, Ghana. 2024).

First Trimester	Second Trimester	Third Trimester
Spontaneous abortion Missed abortion Exhaustion Altered appetite Breast pain Yeast infections Weight loss Headache Nausea and vomiting Pica	Spontaneous abortion Premature contraction Weight gain Dizziness Headaches Yeast infections Hemorrhoids Backache Fluid retention Difficult sleep pattern Leg cramps Joint pain Hair loss	Premature contractions Abruption placentae dehydration Heartburn Indigestion Yeast infections Fluid retention Dizziness Hemorrhoids Constipation Backache Difficult sleep pattern Discomfort while sleeping Increased urination

Source [14].

In Africa, rising temperatures are anticipated to enhance the transmission and spread of vector-borne diseases by increasing mosquito density in specific regions and amplifying the replication rate and frequency of mosquito bites [26]. Furthermore, the increased sweating leading to dehydration as part of thermoregulation in pregnant women can potentially trigger premature labor onset and protract the duration of labor [15]. A study conducted by Costello et al. [26] contends that malaria, dengue fever, and tick-borne encephalitis are poised to become more prevalent, putting individuals without current infections or lacking sufficient immunity at risk in the future. Additionally, there is a heightened potential for increased schistosomiasis infections [16]. Climate change intensifies mercury (Hg) exposure through its impact on environmental conditions and ecosystems. Rising temperatures increase the conversion of elemental mercury into toxic methylmercury in aquatic systems, which accumulates in fish and other organisms at the top of the food chain. Warming waters, such as in the Gulf of Maine, have already been linked to significant increases in methylmercury levels in species like tuna, even as overall mercury emissions decrease. This process poses heightened health risks for populations consuming seafood, as methylmercury is a potent neurotoxin [27]. Additionally, altered precipitation patterns and thawing permafrost caused by climate change release stored mercury into water systems, further amplifying exposure risks in regions like the Arctic. Mitigation strategies require addressing both mercury emissions and greenhouse gas reductions to curb the compounding effects of climate change on mercury contamination [17].

Heat stress during heat events can lead to adverse effects on maternal and perinatal health, including the neonatal period. Air pollution increases the risk of infants being born with low birth weight and experiencing preterm birth, which is the leading cause of neonatal mortality globally. The lack of access to clean energy significantly contributes to climate change and negatively impacts maternal and newborn health by increasing exposure to harmful pollutants and limiting access to essential healthcare services [28]. For example, Lack of clean energy often forces reliance on traditional biomass fuels (e.g., wood, charcoal, dung) and fossil fuels (e.g., coal, kerosene) for cooking, heating, and lighting. This contributes to greenhouse gas emissions, exacerbating climate change through deforestation and carbon dioxide release [29]. In addition to clarifying this, burning biomass fuels and kerosene releases harmful indoor air pollutants, such as PM2.5 and carbon monoxide, linked to preterm births, low birth weight, and stillbirths. Pregnant women and infants are particularly vulnerable, with air pollution affecting fetal development and increasing respiratory illnesses. Limited clean energy also hampers healthcare by restricting essential equipment use and proper vaccine storage, further jeopardizing maternal and neonatal health [30]. Climate change indirectly impacts vulnerable populations through various mechanisms, potentially resulting in profound social, economic, and health implications [31]. Disruptions in services from the COVID-19 pandemic could lead to additional maternal and under-five child fatalities. Contaminant exposure during the prenatal period may impact cardiovascular homeostasis, potentially increasing the burden of diseases attributable to contaminant exposure [20].

Lead exposure has been linked to neurodevelopment and behavior in children, with recent studies revealing a correlation between cord blood lead concentrations and inattention, even at concentrations below 10 mg/L [20]. In Arctic Russia, associations have been noted between spontaneous abortions and mercury levels in the blood, but no adverse associations were identified between maternal exposure to nickel and the risk of delivering a newborn with genital organ malformations [32].

The global communities focused on health and climate change must collaborate and mobilize initiatives to heighten awareness among policymakers about the repercussions of climate change on the health of women and children, as well as on forthcoming generations [33]. It is essential to enhance the availability of high-quality data regarding the influence of climate change on maternal and newborn health. This improvement aims to comprehend the global burden and its attributes, particularly in the most vulnerable and least resilient societies that are currently and will be most significantly impacted by climate change [34].

### Policy Implications to Minimize the Negative Impacts of Climate Change on Maternity and Child Health

Maintaining a healthy diet is crucial for preventing infections, lowering infant mortality, and managing obesity and chronic illnesses [28]. Climate change impacts food production, contributing 20%–30% of greenhouse gas emissions. It may also raise the hazards of weather-related disasters to the health of newborns, making child health a priority in the adaptation process [28]. To mitigate this effect, policies addressing food security with climate change and its impact on mothers and children are needed. Fair distribution of food production and monitoring of nutritional content are essential. Sustainable environmental policies should support clean, sustainable energy, water access, and natural resource management [32].

Gender inequality in low- and middle-income countries should be addressed by empowering women and girls to make decisions, facilitate access to resources and basic services, and support them during climate-related disasters [35]. Enhanced policies and actions to improve disaster preparedness, such as coordinated emergency planning and assistance for women, are also necessary. Breastfeeding infants can be challenging due to climate change events, but it supports policy while reducing greenhouse gas emissions [28, 32].

The disproportionate impact of climate change on women’s health and food security is not given enough priority in the UN and national agendas. Women and children are more susceptible to its effects due to changes in their anatomy, physiology, and sociocultural background [31, 35]. To minimize the effect on maternal and child health, behavioral change policies, health education about heat-related hazards, natural ways to lessen urban heat islands, space cooling in medical facilities, and fair changes in food and housing systems are effective ways to lower the dangers associated with heat [31].

In Africa, the effects of climate change on mother and newborn health will likely continue to be severe. Research is needed to determine who is most at risk, close knowledge gaps, and coordinate initiatives to lessen detrimental effects on health. For adaptation programs to be more effective and widely adopted, children and young people must be meaningfully included in their design and execution [34, 36].

### Future Directions

Future research on integrating maternal and child health into climate change mitigation should focus on understanding the direct and indirect impacts of climate change on maternal and child health, particularly in vulnerable populations and low-resource settings. Adaptive healthcare models need to be developed to address climate-induced risks such as malnutrition, infectious diseases, and heat stress in pregnant women and children. Studies should assess the effectiveness of integrating climate resilience strategies into existing maternal and child health programs while exploring emerging technologies to monitor and mitigate these effects.

Research must evaluate the long-term consequences of prenatal exposure to climate-related pollutants on child development and health outcomes. Cost-effective interventions aimed at reducing maternal and neonatal mortality during climate-related disasters are critical. Additionally, the psychosocial impacts of climate change on maternal mental health and its influence on child-rearing practices require attention. The intersection of water scarcity, hygiene, and maternal-child health in climate-stressed regions should be explored, alongside the design of community-based education programs to raise awareness of climate risks on health.

Innovative solutions, such as climate-smart healthcare facilities, can be tested to simultaneously reduce carbon emissions and improve care quality. Research should also address disparities in health outcomes caused by socioeconomic inequalities and identify policies that integrate sustainable agriculture with nutrition-focused maternal and child health initiatives. The role of indigenous knowledge in mitigating climate impacts on maternal and child health deserves exploration. Finally, modeling future scenarios of climate change effects on maternal and child health can guide policy decisions, with interdisciplinary collaboration to establish evidence-based guidelines for integrating climate action into maternal and child health frameworks.

## Discussions

### Research Limitations

Climate change negatively affects life support systems, including air, food, water, shelter, and security, on which humans depend [37]. Climate change negatively impacts regions with development constraints, disproportionately affecting women and children. It leads to acute food insecurity, reduced water security, heat stress, extreme weather events, and air pollution [37]. Annually, it causes around 2.0 million stillbirths, 2.5 million neonatal deaths, and 295,000 maternal deaths, primarily in low-income countries [38]. This segment identifies some areas that could advance our understanding of complex climate-related phenomena, guide effective decision-making, and develop strategies to mitigate and adapt to the impacts of climate change [26].

Research is needed to assess the effects of climate change and heat waves on maternal and perinatal health in low- and middle-income countries. This will help develop targeted interventions and strategies to improve health outcomes [26]. Replicating and intensifying efforts like the CHAMNHA (Climate, Heat, and Maternal and Neonatal Health in Africa) project can help tailor intervention strategies and review global policies. Investigating lags in adopting low-emission technologies in developing countries is essential for policy recommendations and capacity-building initiatives [39, 40]. Research should also focus on mitigation strategies like energy efficiency, improved forest management, and reduced food waste and loss. Understanding these measures within a holistic framework is crucial for developing comprehensive climate action plans [40].

Climate change is expected to have the most significant impact on children and future generations, with over 88% of the global disease burden occurring in children under five [33, 34]. Therefore, comprehensive climate change and health research is crucial, particularly in Africa. Research should explore the opportunities and challenges of involving women, children, and young people in climate change policy, as they are often underrepresented in political structures [33]. Research should identify cost-effective and sustainable measures to address climate change to equip children and their communities with the skills and resources to cope with climatic changes, particularly in vulnerable developing countries.

Interdisciplinary collaboration is essential, and key areas for investigation include communication, knowledge transfer, community engagement, capacity-building programs, and inclusivity [34]. Also, addressing the intersection of social and political unrest and climate change’s negative effects on maternal and child health could be a potential research gap in Africa [33]. Studies could investigate healthcare access for pregnant women and children during unrest, as well as how disruptions to agricultural activities, displacement, and economic instability may contribute to malnutrition and related health problems [34].

### Conclusion

The review presents a comprehensive framework that combines health policies, environmental sustainability efforts, and strategies for enhancing community resilience to address maternal and child health challenges. It highlights the potential benefits of combining health initiatives with climate change mitigation, guiding policymakers and healthcare practitioners in formulating integrated strategies for sustainable development. The study also advocates for a paradigm shift towards inclusive and interdisciplinary solutions, acknowledging the inextricable linkages between human health and environmental wellbeing in the face of a changing climate.
